# Can Mindfulness Help to Alleviate Loneliness? A Systematic Review and Meta-Analysis

**DOI:** 10.3389/fpsyg.2021.633319

**Published:** 2021-02-25

**Authors:** Siew Li Teoh, Vengadesh Letchumanan, Learn-Han Lee

**Affiliations:** ^1^School of Pharmacy, Monash University Malaysia, Selangor, Malaysia; ^2^Novel Bacteria and Drug Discovery (NBDD) Research Group, Microbiome and Bioresource Research Strength, Jeffrey Cheah School of Medicine and Health Sciences, Monash University Malaysia, Selangor, Malaysia

**Keywords:** mindfulness, loneliness, systematic review, meta-analysis, randomized controlled trial

## Abstract

**Objective:** Mindfulness-based intervention (MBI) has been proposed to alleviate loneliness and improve social connectedness. Several randomized controlled trials (RCTs) have been conducted to evaluate the effectiveness of MBI. This study aimed to critically evaluate and determine the effectiveness and safety of MBI in alleviating the feeling of loneliness.

**Methods:** We searched Medline, Embase, PsycInfo, Cochrane CENTRAL, and AMED for publications from inception to May 2020. We included RCTs with human subjects who were enrolled in MBI with loneliness as an outcome. The quality of evidence was assessed using Cochrane's Risk of Bias (ROB) tool and Grading of Recommendations Assessment, Development, and Evaluation (GRADE). A random-effects model was used for meta-analysis.

**Results:** Out of 92 articles identified, eight studies involving 815 participants were included in this study. Most (7/8) trials conducted a minimum of 8 weeks of MBI. Most of the trials (5/8) used UCLA-Loneliness Scale. A pooled analysis combining three trials and compared with wait-list showed significant improvement in loneliness score reduction using the UCLA-R scale with MD of −6.33 [95% confidence interval (CI): −9.39, −3.26]. Subgroup analysis with only two Cognitively-Based Compassion Training (CBCT) trials also showed similar MD of −6.05 (95% CI: −9.53, 2.58). The overall quality of evidence (GRADE) was low.

**Conclusions:** Mindfulness intervention with an average length of 8-week duration significantly improved the population's loneliness level with no mental health issue. However, this evidence had a low GRADE level.

## Introduction

Loneliness is defined as a perceived discrepancy between the desired and the attained social relationships (Paloutzian et al., 1982). A recent study in 2020 showed a high prevalence of loneliness in the USA, with 13.8% of adults felt that they were always or often lonely (McGinty et al., 2020). Similarly, a high prevalence of loneliness has also been found in Europe and Asia, with 15.6–49.3% of the populations often feeling lonely or were at risk of social isolation (Yang and Victor, 2008; Ibrahim et al., 2013; Nyqvist et al., 2017). Moreover, loneliness is associated with various diseases. A systematic review of observational studies found that those with poor social relationships have an increased risk of coronary heart disease and stroke by 29 and 32%, respectively (Valtorta et al., 2016). Besides, loneliness has also been well-established as one of the risk factors for mortality, with an estimated increased risk of 29–32% (Holt-Lunstad et al., 2015).

Many studies (Creswell et al., 2012; Jazaieri et al., 2012; Dodds et al., 2015; Mascaro et al., 2018; Zhang et al., 2018; Lee et al., 2019; Lindsay et al., 2019; Pandya, 2019) have found an effect of mindfulness-based intervention in alleviating loneliness. Originated from the Buddhist meditation system and now being widely applied in the clinical setting world (Bodhi, 2011), mindfulness is defined as the awareness that emerges through paying attention to the present moment with a nonjudgmental attitude (Kabat-Zinn, 2003). Mindfulness practices have been shown to foster vigilance, improve communication and empathy, and improve mental and physical health (Brown et al., 2007).

Several studies have evaluated the effectiveness of mindfulness intervention to alleviate loneliness (Creswell et al., 2012; Jazaieri et al., 2012; Dodds et al., 2015; Mascaro et al., 2018; Zhang et al., 2018; Lee et al., 2019; Lindsay et al., 2019; Pandya, 2019). However, there is a lack of critical appraisal and summary of these studies. Previous systematic reviews evaluated the effect of mindfulness intervention on different outcomes, e.g., in depression and anxiety (Zhang et al., 2015; Zou et al., 2018) and pain (Hilton et al., 2017). Moreover, these reviews only focused on populations with physical or mental illness (Zhang et al., 2015; Hilton et al., 2017; Zou et al., 2018). There was no systematic review that summarized the current findings on mindfulness's effects in alleviating loneliness in populations that are either healthy or with medical conditions. Therefore, this systematic review aimed to critically synthesis the evidence of current clinical trials in alleviating loneliness in all populations.

## Methods

This systematic review was performed in accordance with the principles outlined in the Cochrane Handbook for Systematic Reviews of Interventions (Higgins et al., 2019) and is reported with the Preferred Reporting Items for Systematic Review and Meta-analysis (PRISMA) (Moher et al., 2009). The review protocol is registered with PROSPERO (registration no. 170238).

### Search Strategy and Study Selection

Five databases were used to search for relevant articles, including Medline, Embase, PsycINFO, Cochrane Central Register of Controlled Trials (CENTRAL), and Allied and Complementary Medicine (AMED). The search strategy used was the combination of related keywords of “mindfulness” and “loneliness,” e.g., (loneliness OR lonel^*^ OR UCLA Loneliness Scale) and (mindfulness OR meditation or transcendental meditation). The complete search strategy was reported in Supplementary Table 1. Studies to be included must be (1) randomized controlled trial, (2) which recruited human participants (of any age, with or without any health condition) intervened with any intervention of mindfulness, (3) compared with a group without mindfulness component, and (4) with the assessment on loneliness.

### Data Extraction

Two authors (SLT and VL) working independently used a standardized data extraction sheet to extract the trials' characteristics and results. Any disagreement was resolved by discussion to reach a consensus. A third author's opinion (LHL) was sought after when needed. The authors extracted the information on study design, blinding status, participants' characteristics, interventions, comparators, clinical assessment, and the outcomes at baseline and postintervention. The clinical evaluation related to loneliness was the primary outcome. Besides, any adverse effect reported in the trials was considered the secondary outcome of interest.

### Study Quality Assessment

The methodological quality of each trial was assessed by two independent reviewers (SLT and VL) using the Cochrane Risk of Bias (ROB) Tool 2.0 (Higgins et al., 2011; Sterne et al., 2019). The methodological evaluation domains included randomization, the effect of adhering to intervention, missing outcome data, outcome, and selection of the reported result (Higgins et al., 2011; Sterne et al., 2019). The funding of the trials was assessed within the domain of “other sources of bias.” Each trial was classified as having low risk (low ROB for all domains), high risk (high ROB for 1 or more domains), or some concerns (some concerns for one or more key domains, given no high ROB in any domain) (Higgins et al., 2011; Sterne et al., 2019).

### Data Analysis

The results were expressed as mean differences (MDs) with 95% confidence intervals (CIs) to determine the effect of mindfulness on loneliness and a continuous outcome. The change from baseline was compared between the mindfulness group and the comparator group. Data from trials measured using the same loneliness scales were pooled in a meta-analysis and expressed as MDs, using an inverse-variance method with a random-effects model (DerSimonian and Laird, 1986). Data from trials measured using similar loneliness scales were pooled in a meta-analysis using standardized mean difference (SMD). Using SMD, 0.20 indicated a small effect, 0.50 a moderate effect, and 0.8 a large effect (Cohen, 2013). The heterogeneity of the included trials was assessed using the chi-squared test and the *I*^2^ test. For the chi-squared test, *p* ≤ 0.10 indicated statistically significant heterogeneity (Higgins et al., 2019). An *I*^2^ value of more than 50% revealed substantial heterogeneity (Higgins et al., 2019). Subgroup analysis and explorative analysis were performed to add or remove any heterogeneity in participants, interventions, comparators, and outcome measurements.

For a meta-analysis with at least 10 trials included, publication bias was assessed using Egger's test (Egger et al., 1997) to calculate the significance level of funnel plot asymmetry, where *p* < 0.10 indicates significant funnel plot asymmetry (Sterne et al., 2011). The software used for data analysis was Stata version 14 (StataCorp; College Station, Texas, USA).

### Quality of Evidence

The overall quality of evidence was assessed independently by two authors (SLT and VL) based on the domains of study design, ROB of individual trials, heterogeneity, the directness of evidence, precision of effect estimates, and possibility of publication bias, using the Grading of Recommendations Assessment, Development, and Evaluation (GRADE) approach (Andrews et al., 2013). The overall quality of evidence ranged from high, moderate, low to very low. The high quality indicates a high degree of certainty that the estimated effect lies close to the true effect. In contrast, low quality means substantial uncertainty about the estimated impact (Guyatt et al., 2008).

## Results

**Figure 1:**
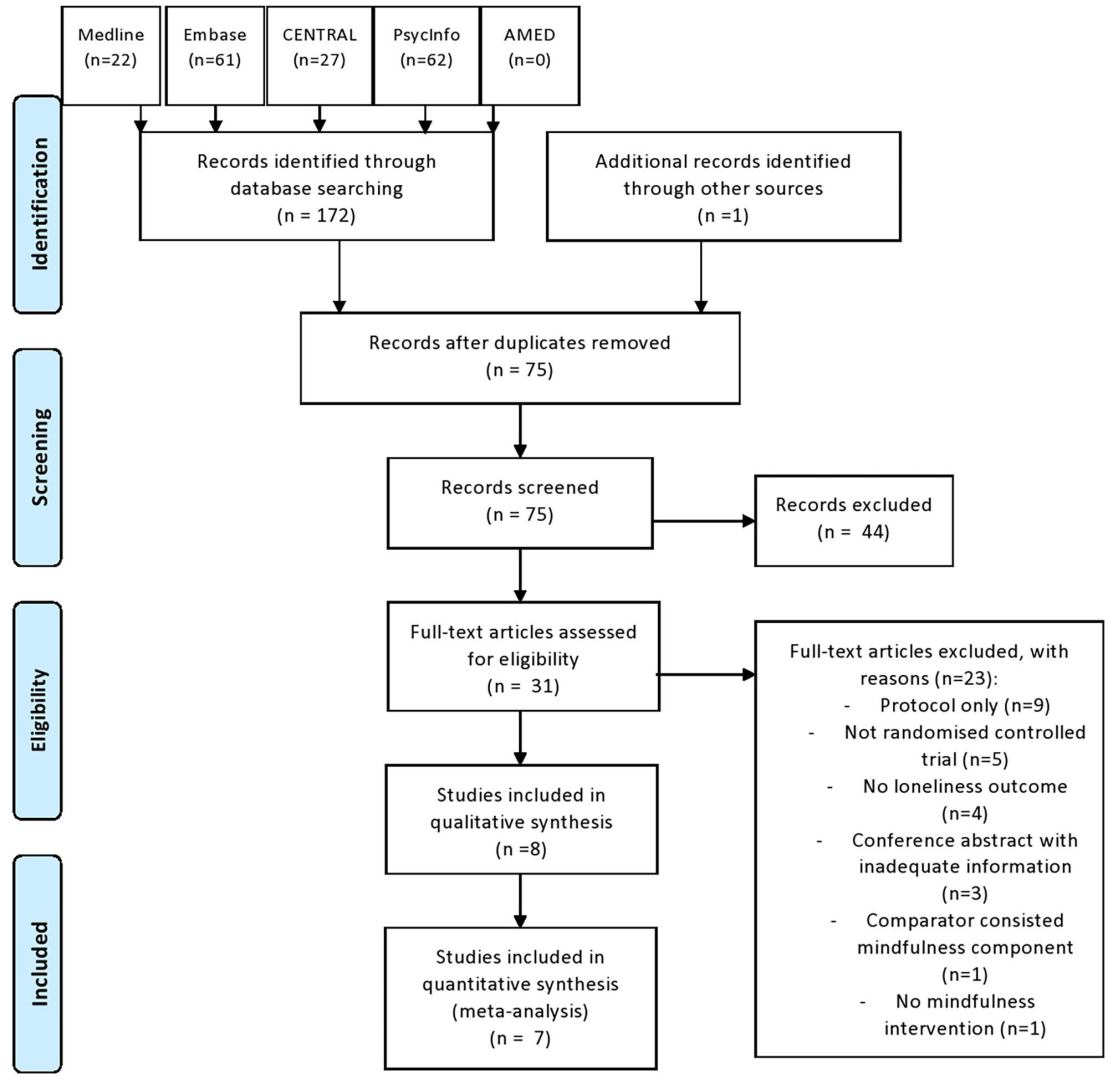
Flow of study selection.

The search yielded 189 articles: 188 identified from electronic databases and one obtained by bibliography search. A total of 97 duplicates were removed. Of the remaining 92 studies screened, only 31 were relevant and were retrieved for full-text review. The full-text review revealed only eight studies that met the inclusion criteria. The 23 excluded studies were protocol (*n* = 9), not randomized controlled trial (*n* = 5), no loneliness outcome (*n* = 4), conference abstract with inadequate information (*n* = 3), comparator-consisted mindfulness component (*n* = 1), and no mindfulness intervention (*n* = 1). This review included eight trials involving 815 participants. Figure 1 shows the flow diagram of this study selection.

### Study Characteristics

Table 1 summarizes the characteristics of the eight included trials (Creswell et al., 2012; Jazaieri et al., 2012; Dodds et al., 2015; Mascaro et al., 2018; Zhang et al., 2018; Lee et al., 2019; Lindsay et al., 2019; Pandya, 2019). The trials were conducted in the USA (*n* = 6) (Creswell et al., 2012; Jazaieri et al., 2012; Dodds et al., 2015; Mascaro et al., 2018; Zhang et al., 2018; Lindsay et al., 2019), India (*n* = 1) (Pandya, 2019), and Korea (*n* = 1)(Lee et al., 2019).

**Table 1:** Characteristics of included studies.

**Author (year)**	**Country**	**Condition; Age (years)**	**Mental or cognitive functions**	***n* (ITT); n (PP)**	**Intervention**	**Description of Intervention**	**Frequency**	**Duration**	**At-home practice**	**Comparator**	**Loneliness scale**
Creswell et al. (2012)	USA	Healthy elderly; 55–85 years (M = 65, SD = 7)	No dementia (according to MMSE score of 27–28)	40; 34	MBSR	Group sessions consist of guided mindfulness meditation exercises, mindful yoga and stretching, and group discussions with the intent to foster mindful awareness of one's moment-to-moment experience. A day-long retreat in the sixth or seventh week.	Once weekly 2 h	8 weeks	Yes−30 min daily practice	Wait-List	UCLA-R
Jazaieri et al. (2012)	USA	Adults (M = 32.87, SD = 8.83 [(Intervention); M = 32.88, SD = 7.97 (Control)]	Social anxiety disorder, including some with depression	56; 30	MBSR	Group classes, a 1-day meditation retreat, and daily home practice	Once weekly 2.5 h	3 months	Yes—daily (time NS)	Aerobic exercise	UCLA-8^*^
Dodds et al. (2015)	USA	Women with history of breast cancer; [M = 54.7, SD = 12.1 (Intervention); M = 55.8, SD = 9.7 (Control)]	No obvious condition (according to scales of depression, stress and mental well-being)	33; 28	CBCT	Group sessions consist of classes through didactics, class discussion, and guided meditation practice.	Once weekly 2 h	8 weeks	Yes—at least 30 min practice three times weekly	Wait-List	UCLA-R
Mascaro et al. (2018)	USA	Medical students; 22-30 (M = 25, SD = 1.89)	No obvious condition (according to scales of depression)	59; 32	CBCT	A sequence of 10 classes included didactic teaching combined with meditations	Once weekly 1.5 h	10 weeks	Yes−20 min daily	Wait-list	UCLA-R
Zhang et al. (2018)	USA	Chinese college students; 17–25	Elevated loneliness level (claimed by author)	50; 41	MBCT	Derived from MBSR and designed for people with a history of recurrent depression to help prevent future recurrences.	Once weekly 2 h	8 weeks	Yes— (details NS)	Not stated	Indigenous loneliness test
						On-campus group sessions adapted by substituting the depression-related information with loneliness psychoeducation.					
Lee et al. (2019)	Korea	Adults with hypertension or/and type-2 diabetes [M = 67.88, SD = 4.95 (Intervention); M = 69.55, SD = 7.22 (Control)]	Not reported	46; 35	BEM	A series of yoga-like exercises	Twice weekly of 75 min	8 weeks	NS	Health education class	Loneliness score as part of mental health test
Lindsay et al. (2019)	USA	Adults with stress (M = 32, SD = 14)	Elevated stress level	153; 93	14-lesson, smartphone-based interventions	Mindfulness meditation which involved monitoring present-moment experiences with an orientation of acceptance	Daily 20 min	2 weeks	Yes−3-10 min daily	Guidance in free reflection, analytic thinking, and problem solving without mindfulness content	UCLA-R
Pandya (2019)	India	Elderly (Retired 2–5 years); 62–68	Probable depression/low mental well-being (according to WEMWBS scale)	378; 323	Yoga	Lessons consisted of meditation, asanas (yoga poses) and relaxation.	Once weekly 45 min	2 years	Yes—once a week (time NS)	No intervention	De Jong Gierveld Loneliness Scale

*BEM, Brain Education-based Meditation; CBCT, Cognitively-Based Compassion Training; ITT, Intention-To-Treat; M, Mean; MBCT, Mindfulness-based cognitive therapy; MBSR, Mindfulness-Based Stress Reduction; MMSE, Mini-Mental State Examination; NS, Not Stated; PP, Per Protocol; SD, Standard Deviation; WEMWS, Warwick-Edinburgh Mental Wellbeing Scale*.

* *UCLA-8 Loneliness Scale is a short version of UCLA-R Loneliness Scale*.

The sample size of the trials was generally small, ranging from 33 participants (Dodds et al., 2015) to 153 (Lindsay et al., 2019) participants, except for one trial (Pandya, 2019) with a relatively bigger sample size of 378 participants. Two trials recruited younger populations who were students (Mascaro et al., 2018; Zhang et al., 2018), while the other six trials were adults (Creswell et al., 2012; Jazaieri et al., 2012; Dodds et al., 2015; Lee et al., 2019; Lindsay et al., 2019; Pandya, 2019). One trial recruited only women with a history of breast cancer (Dodds et al., 2015), while the other seven trials recruited both genders (Creswell et al., 2012; Jazaieri et al., 2012; Mascaro et al., 2018; Zhang et al., 2018; Lee et al., 2019; Lindsay et al., 2019; Pandya, 2019). In terms of mental health conditions, one trial recruited participants with a social anxiety disorder (Jazaieri et al., 2012), one trial with depression (Pandya, 2019), one trial with elevated stress level (Lindsay et al., 2019), and one trial with elevated loneliness level (Zhang et al., 2018). Three trials recruited participants with no obvious mental or cognitive conditions (Creswell et al., 2012; Dodds et al., 2015; Mascaro et al., 2018), and one trial did not report the characteristics of included participants (Lee et al., 2019).

The intervention of two trials was mindfulness-based stress reduction (MBSR) (Creswell et al., 2012; Jazaieri et al., 2012), two cognitively based compassion training (CBCT) (Dodds et al., 2015; Mascaro et al., 2018), one mindfulness-based cognitive therapy (MBCT) (Zhang et al., 2018), one brain education-based meditation (BEM) (Lee et al., 2019), one mindfulness meditation with an orientation of acceptation (Lindsay et al., 2019), and one yoga (Pandya, 2019). Almost all of the interventions (7/8) (Creswell et al., 2012; Jazaieri et al., 2012; Dodds et al., 2015; Mascaro et al., 2018; Zhang et al., 2018; Lee et al., 2019; Pandya, 2019) were conducted as a group session and one (Lindsay et al., 2019) smartphone-based intervention. All interventions consisted of guided meditations. However, for MBSR (Creswell et al., 2012; Jazaieri et al., 2012), MBCT (Zhang et al., 2018), and CBCT (Dodds et al., 2015; Mascaro et al., 2018), they consisted of the addition of yoga and stretching, group discussions, and a day-long retreat. MBSR (Creswell et al., 2012; Jazaieri et al., 2012) and MBCT (Zhang et al., 2018) also consisted of a day-long retreat. Notably, BEM (Lee et al., 2019) and Yoga (Pandya, 2019) had the additional exercise or stretching component in addition to meditation. More than half of the interventions (5/8) (Creswell et al., 2012; Jazaieri et al., 2012; Dodds et al., 2015; Mascaro et al., 2018; Zhang et al., 2018) carried out once-weekly sessions for around 2 h, except one with once weekly for 45 min (Pandya, 2019), one with twice weekly of 75 min (Lee et al., 2019), and one with once daily of 20 min (Lindsay et al., 2019). Almost all (7/8) interventions (Creswell et al., 2012; Jazaieri et al., 2012; Dodds et al., 2015; Mascaro et al., 2018; Zhang et al., 2018; Lee et al., 2019; Pandya, 2019) took at least 8 weeks with one with a longer duration of 2 years (Pandya, 2019), and only one of 2 weeks (Lindsay et al., 2019).

Three trials (Creswell et al., 2012; Dodds et al., 2015; Mascaro et al., 2018) used wait-list (i.e., control group's participants were placed on a wait-list while the trial was ongoing) as a comparator. Three other trials used active control group which included aerobic exercise (Jazaieri et al., 2012), health education class (Lee et al., 2019), and guidance in reflective thinking and problem solving without mindfulness content (Lindsay et al., 2019). One trial (Pandya, 2019) assigned no intervention to the comparator group, while the other trial (Zhang et al., 2018) did not mention the comparator type.

### Quality Assessment

Based on the assessment using Cochrane's ROB tool version 2.0, almost all trials (7/8) (Creswell et al., 2012; Jazaieri et al., 2012; Dodds et al., 2015; Mascaro et al., 2018; Zhang et al., 2018; Lee et al., 2019; Pandya, 2019) had high ROB and one trial (Lindsay et al., 2019) with some concerns (Table 2). The trial had some concerns in randomization components with low ROB for all other components.

**Table 2:** Quality assessment of trials using risk of bias tool.

**Trials**	**Randomization**	**Effect of adhering to intervention**	**Missing outcome data**	**Outcome**	**Selection of the reported result**	**Overall**
Creswell et al. (2012)	Some concerns	High	Low	Low	Some concerns	High
Jazaieri et al. (2012)	Some concerns	High	Low	Low	Some concerns	High
Dodds et al. (2015)	Some concerns	High	Low	Low	Some concerns	High
Mascaro et al. (2018)	Some concerns	High	Low	Low	Some concerns	High
Zhang et al. (2018)	Some concerns	High	Low	Low	Some concerns	High
Lee et al. (2019)	Low	High	Low	Low	Some concerns	High
Lindsay et al. (2019)	Some concerns	Low	Low	Low	Low	Some concerns
Pandya (2019)	Some concerns	High	Low	Low	Some concerns	High

In the randomization component, generally appropriate sequence randomization was used. However, almost all trials (6/8) (Creswell et al., 2012; Jazaieri et al., 2012; Dodds et al., 2015; Mascaro et al., 2018; Zhang et al., 2018; Pandya, 2019) most likely did not conceal the allocation while one trial (Lindsay et al., 2019) did not specify and only one trial (Lee et al., 2019) mention about the appropriate randomization concealment.

For the effect of adhering to intervention, only one trial had low ROB (Lindsay et al., 2019), while the rest (7/8) had some concerns in nonadherence to the assigned intervention regimen that could have affected participants' outcomes. The trial with low ROB (Lindsay et al., 2019) provided raw data for baseline and postintervention for all patients, including those who have dropped out, to enable an appropriate analysis to estimate the effect of adhering to an intervention.

All trials had low ROB for missing outcome data, with outcome data available for all or nearly all participants randomized. All trials also had ROB for outcome domain, as all trials have used appropriate and only objective assessment (loneliness measurement scale). For the selection of the reported results, only one trial (Lindsay et al., 2019) had pre-specified and registered protocol while other trials (7/8) (Creswell et al., 2012; Jazaieri et al., 2012; Dodds et al., 2015; Mascaro et al., 2018; Zhang et al., 2018; Lee et al., 2019; Pandya, 2019) did not mention the availability of any pre-specified analysis plan. The details of the ROB assessment were available in Supplementary Table 2.

### Effects of Mindfulness in Alleviating Loneliness

Two established loneliness scales were employed, with UCLA loneliness scale being the most common scale used to measure loneliness in five trials (Creswell et al., 2012; Jazaieri et al., 2012; Dodds et al., 2015; Mascaro et al., 2018; Lindsay et al., 2019). The other trials used De Jong Gierveld Loneliness Scale (Pandya, 2019), mental health test with a loneliness component (Lee et al., 2019), and a loneliness test in the author's indigenous language (Zhang et al., 2018). All except one trial used scales that indicated higher score with more loneliness (including UCLA loneliness scale) while the exceptional trial used scale showed lower scores with more loneliness (i.e., De Jong Gierveld Loneliness Scale).

Referring to the effects of individual trials as shown in Table 3, half of the trials (4/8) (Creswell et al., 2012; Mascaro et al., 2018; Zhang et al., 2018; Pandya, 2019) showed significant loneliness reduction after mindfulness intervention compared with the comparator group, and they shared the common feature of the intervention of at least 8 weeks. Another half of the trials (Jazaieri et al., 2012; Dodds et al., 2015; Lee et al., 2019; Lindsay et al., 2019) did not show significant changes.

**Table 3:** Effect size of mindfulness intervention in improving loneliness.

**Trials**	**Scale**	**Range of score of scale**	**Mean difference (95% CI)**
Creswell et al. (2012)	UCLA-R	20–80	−7.30 (−13.81, −0.79)^*^
Jazaieri et al. (2012)	UCLA-8	0–100^~^	−0.79 (−3.74, 2.16)
Dodds et al. (2015)	UCLA-R	20–80	−2.40 (−12.01, 7.21)
Mascaro et al. (2018)	UCLA-R	20–80	−6.50 (−10.20, −2.80)^*^
Zhang et al. (2018)	Indigenous loneliness test	NA (unable to retrieve article)	−4.77 (−8.60, −0.94)^*^
Lee et al. (2019)	Loneliness score as part of mental health test	1–5 (Likert scale)	0.03 (−0.70, 0.76)^∧^
			−0.17 (−0.87, 0.53)
Lindsay et al. (2019)	UCLA-R	20–80	1.60 (−3.11, 6.29)
Pandya (2019)^@^	De Jong Gierveld Loneliness Scale	5–35	2.41 (2.20, 2.62)^*^

@ *Lower score indicated more loneliness; while for all other trials, higher score indicated more loneliness*.

~ *Obtained from original literature of the scale (Hays and DiMatteo, 1987)*.

* *Statistically significant result*.

∧ *Conservative estimate from Lee et al. (2019)*.

Referring to Table 4, the main pooled analysis combining three trials in participants with no known mental health conditions, which employed slightly varied mindfulness interventions (i.e., two CBCT and one MBSR) and compared with wait-list, showed significant improvement in loneliness score reduction using UCLA-R scale with MD of −6.33 (95% CI: −9.39, −3.26), *I*^2^ = 0.0%, *p* = 0.688; three trials; Grade low) (Supplementary Figure 1). There was no significant publication bias using Egger's test (*p* = 0.602). However, this value was only indicative owing to the small number of included studies in the meta-analysis. A subgroup analysis with only CBCT intervention, with the removal of one trial which employed MBSR, also showed similar results of MD of −6.05 (95% CI: −9.53, 2.58, *I*^2^ = 0.0%, *p* = 0.425; two trials; Grade low) (Supplementary Figure 2). A subgroup analysis with only participants with mental health conditions found no significant improvement with small effect in loneliness score reduction using varied scales with SMD of −0.23 (95% CI: −0.80, 0.33), *I*^2^ = 62.8%, *p* = 0.068; three trials; Grade very low) (Supplementary Figure 3). Another subgroup analysis comparing younger populations with adults and elderly showed significant improvement with large effect only in younger populations with SMD of −0.85 (95% CI: −1.36, −0.35), *I*^2^ = 0.0%, *p* = 0.751; two trials; Grade low) (Supplementary Figure 4). No significant improvement with small effect in loneliness score reduction was found in adults and elderly with SMD = −0.12 (95% CI: −0.43, 0.19), *I*^2^ = 18%, *p* = 0.300; five trials; Grade low) (Supplementary Figure 5) or SMD = −0.15 (95% CI: −0.46, 0.15), *I*^2^ = 15.6%, *p* = 0.315; five trials; Grade low) (Supplementary Figure 6). Two pooled estimates were available as two estimates were reported from one trial with loneliness-related questions. Table 5 explains in detail on the GRADE.

**Table 4:** Pooled analysis of mindfulness intervention in improving loneliness.

**Type of analysis (PICO)**	**Trials**	**Pooled Mean difference (95% CI)**
Main Analysis: P: Varied characteristics (no known mental health conditions) I: Varied mindfulness interventions C: Wait-list O: UCLA-R scale	Creswell et al., 2012 Dodds et al., 2015 Mascaro et al., 2018	MD = −6.33 (−9.39, −3.26)^*^ *I*^2^ = 0.0%, *p* = 0.688
Subgroup Analysis 1 (CBCT only): P: Varied characteristics (no known mental health conditions) I: CBT only^~^ C: Wait-list O: UCLA-R scale	Dodds et al., 2015 Mascaro et al., 2018	MD = −6.05 (−9.53, −2.58)^*^ *I*^2^ = 0.0%, *p* = 0.425
Subgroup Analysis 2 (Participants with mental health conditions): P: Participants with mental health conditions^~^ I: Varied mindfulness interventions C: Varied comparators^~^ O: Varied scales^~^	Jazaieri et al., 2012 Zhang et al., 2018 Lindsay et al., 2019	SMD = −0.23 (−0.80, 0.33) *I*^2^ = 62.8%, *p* = 0.068
Subgroup Analysis 3.1 (Younger populations only): P: Younger populations only^~^ I: Varied mindfulness interventions C: Varied comparators^~^ O: Varied scales^~^	Mascaro et al., 2018 Zhang et al., 2018	SMD = −0.85 (−1.36, −0.35)^*^ *I*^2^ = 0.0%, *p* = 0.751
Subgroup Analysis 3.2 (Adults and elderly only): P: Adults and elderly only^~^ I: Varied mindfulness interventions C: Varied comparators^~^ O: Varied scales^~^	Creswell et al., 2012 Jazaieri et al., 2012 Dodds et al., 2015 Lee et al., 2019 Lindsay et al., 2019	SMD = −0.12 (−0.43, 0.19)^@^ *I*^2^ = 18.0%, *p* = 0.300 SMD = −0.15 (−0.46, 0.15) *I*^2^ = 15.6%, *p* = 0.315
Explorative Analysis 1: P: 3 no known mental health conditions, 1 with elevated stress^~^ I: Varied mindfulness interventions C: 3 Wait-list and 1 Active control^~^ O: UCLA-R scale	Creswell et al., 2012 Dodds et al., 2015 Mascaro et al., 2018 Lindsay et al., 2019	MD = −3.74 (−8.45, 0.98) *I*^2^ = 64.3%, *p* = 0.039
Explorative Analysis 2: P: 3 no known mental health conditions, 1 with elevated stress and 1 with SAD^~^ I: Varied mindfulness interventions C: 3 Wait-list and 1 aerobic exercise^~^ O: UCLA-R scale and 1 UCLA-8 scale^~^	Creswell et al., 2012 Jazaieri et al., 2012 Dodds et al., 2015 Mascaro et al., 2018 Lindsay et al., 2019	SMD = −0.33 (−0.76, 0.10) *I*^2^ = 53.6%, *p* = 0.071
Explorative Analysis 3: P: 1 no known mental health conditions and 1 with SAD^~^ I: MBSR only^~^ C: Wait-list O: UCLA-R scale and 1 UCLA-8 scale^~^	Creswell et al., 2012 Jazaieri et al., 2012	SMD = −0.48 (−1.02, 0.06) *I*^2^ = 13.9%, *p* = 0.281
Explorative Analysis 4: P: Varied characteristics (with and without known mental health conditions)^~^ I: Varied mindfulness interventions C: Varied comparators^~^ O: Varied scales^~^	Creswell et al., 2012 Jazaieri et al., 2012 Dodds et al., 2015 Mascaro et al., 2018 Zhang et al., 2018 Lee et al., 2019 Lindsay et al., 2019	SMD=-0.34 (−0.69, 0.01)^@^ *I*^2^ = 48.8%, *p* = 0.068 SMD = −0.36 (−0.70, −0.03)^*^ *I*^2^ = 45.6%, *p* = 0.088

*P, Participant; I, Intervention; C, Comparator; O, Outcome; CBCT, Cognitively-based compassion training; MBSR, Mindfulness-Based Stress Reduction; SAD, Social anxiety disorder*.

~ *Characteristics of PICO which differ from main analysis no.1*.

@ *Conservative estimate from Lee et al. (2019)*.

* *Statistically significant result*.

**Table 5:** Summary of findings of the effects of chia seed in all indications.

**Outcome**	**Anticipated absolute effects (95%CI)**	**No. of participants (no. of studies)**	**Quality of evidence (GRADE^a^)**
MBSR or CBCT for loneliness assessed with UCLA-R Loneliness Scale in participants with no known mental health conditions (Scale from: 20 to 80; follow-up: range of 8–10 weeks)	Mean score difference in intervention group was 6.33 lower (9.39 lower to 3.26 lower)	94 (3 RCTs)	Low^a,b^
CBCT for loneliness assessed with UCLA-R Loneliness Scale in participants with no known mental health conditions (Scale from: 20 to 80; follow-up: range of 8–10 weeks)	Mean score difference in intervention group was 6.05 lower (9.53 lower to 2.58 lower)	60 (2 RCTs)	Low^a,b^
Varied mindfulness intervention for loneliness assessed with varied loneliness scales in participants with mental health conditions (follow-up: range of 2–12 weeks)	Standardized mean score difference in intervention group was 0.23 lower (0.80 lower to 0.33 higher)	164 (3 RCTs)	Very low^a,b,c^
Varied mindfulness intervention for loneliness assessed with varied loneliness scales in younger participants (follow-up: range of 8–10 weeks)	Standardized mean score difference in intervention group was 0.85 lower (1.36 lower to 0.35 lower)	73 (2 RCTs)	Low^a,b^
Varied mindfulness intervention for loneliness assessed with varied loneliness scales in adults and elderly participants (follow-up: range of 2–12 weeks)	Standardized mean score difference in intervention group was 0.12 lower (0.43 lower to 0.19 higher)^@^	220 (5RCTs)	Low^a,b^
	Standardized mean score difference in intervention group was 0.15 lower (0.46 lower to 0.15 higher)		

*CBCT, Cognitively-Based Compassion Training; MBSR, Mindfulness-Based Stress Reduction*.

a *High risk of bias for all trials for the domain of effect of adhering to intervention*.

b *Small sample size*.

c *High heterogeneity*.

@ *Conservative estimate from Lee et al. (2019)*.

Four explorative analyses were conducted by varying some components of the PICO (Table 4). Specifically, in explorative analysis 1, when 1 additional trial which used a slightly different comparator (i.e., aerobic exercise), a nonsignificant MD of −3.74 (95% CI: −8.45, 0.98, *I*^2^ = 64.3%, *p* = 0.039; four trials) (Supplementary Figure 7). In explorative analysis 2, when one trial which utilized UCLA-8 scale (a simplified version of UCLA-R scale) was added to explorative analysis 1, also a nonsignificant SMD of −0.33 (95% CI: −0.76, 0.10, *I*^2^ = 53.6%, *p* = 0.071; five trials) (Supplementary Figure 8) was obtained. Explorative analysis 3, involved two trials that employed MBSR interventions and wait-list control. All three analysis recruited different mental health status (one with no known mental health illness and one with SAD) and employed slightly different loneliness scale (one with UCLA-R and one with UCLA-8), with a pooled nonsignificant SMD of −0.48 (95% CI: −1.02, 0.06; *I*^2^ = 13.9%, *p* = 0.281) (Supplementary Figure 9). In explorative analysis 4, seven trials which utilized the loneliness scales, which showed a similar trend of an increasing score with increase loneliness were pooled. Two pooled estimates were available as two estimates were reported from one trial with loneliness-related questions. The two different estimates produced a nonsignificant SMD of −0.34 (95% CI: −0.69, 0.01; *I*^2^ = 48.8%, *p* = 0.068) (Supplementary Figure 10) and a significant SMD of SMD = −0.36 (95% CI: −0.70, −0.03; *I*^2^ = 45.6%, *p* = 0.088) (Supplementary Figure 11), respectively.

## Discussion

This is the first known systematic review and meta-analysis investigating the effect on loneliness using mindfulness intervention. The review found the potential usefulness of mindfulness in alleviating loneliness, mostly with CBCT mindfulness intervention in participants with no apparent mental health conditions. Further analyses showed that loneliness alleviation was more pronounced in the younger population than adults and elderly people.

A similar result was also found in another previous SRMA, which found small to medium effects of mindfulness intervention in improving pro-social behaviors (Luberto et al., 2018). The review found mindfulness interventions, including MBSR and MBCT, to significantly enhance positive pro-social emotions (Luberto et al., 2018). Many previous studies have demonstrated the negative correlation between loneliness and pro-social behaviors as loneliness has negatively affected one's pro-social behavior and the interaction with others (Salovey et al., 1991; Woodhouse et al., 2012; Zysberg, 2012).

Although the main findings of this review were limited to the population with no mental health issue, a previous systematic review and meta-analysis (SRMA) summarizing the outcomes of CBCT also showed moderate to large effect sizes of CBCT for the treatment of a wide range of psychiatric disorders including depression and anxiety disorder in their respective clinical symptoms (Butler et al., 2006). Several further analyses (as shown in subgroup and explorative analyses in this review) showed the mindfulness's effects in reducing loneliness became nonsignificant when one or more of the differences of the characteristics of mindfulness intervention, comparator, and participants is incorporated into the analyses. As evident in the high heterogeneity, these analyses should be interpreted with caution. However, they served to generate hypothesis for future studies. Future studies should work to verify the effectiveness in improving loneliness when (i) compared with active control [e.g., physical exercise, a different model of mind or cognitive training (with or without mindfulness component)], (ii) compared between other participants' characteristics (with or without mental health illness), (iii) compared between different mode of mindfulness interventions administration (e.g., varied in length of practice, with our without home practice), and (iv) compared between different loneliness measurement scales.

One of the main challenges in researching the effect of mindfulness intervention in clinical trials is the lack of double-blinding procedures (Davidson and Kaszniak, 2015). Arguably, only one of the included trials used an appropriate comparator, which was the guidance in free reflection, analytic thinking, and problem-solving without mindfulness content. Other trials either used wait-list controls, which was controversial in terms of ethical issues and its potential in overestimating treatment effect (Cunningham et al., 2013). Other inappropriate active comparators including exercises and health education do not blind participants adequately. Future studies could incorporate the content of the mentioned appropriate active comparator for a better study design.

Inconsistency of the use of the scales of loneliness measurement was another issue identified in this review. The concept of loneliness is vague and can be differently interpreted among literature (Bolmsjö et al., 2019). Included trials did not clearly define the idea of loneliness and only described the scales used to measure loneliness. Different populations were found to have different notions of loneliness, ranging from feelings of sadness, abandonment, alienation, emptiness, and not connecting with others/the world outside (Bolmsjö et al., 2019). Therefore, much consideration must be made in the appropriateness of scales used in the study's context, especially about the populations studied. At the very least, the validity of the scales used should be considered.

As with any systematic review and meta-analysis, the review is inherent with the original trials' limitations. A major issue is that all trials have a low quality of evidence (all had either some concerns or a high ROB). The overall low quality of evidence based on the GRADE approach indicates that findings should be interpreted with caution. However, the limitations in terms of the quality of evidence are detailed in Tables 2, 5 and Supplementary Table 2. Although the authors did an extensive literature search, and an effort was made to include gray literature, unpublished studies might be missed.

## Conclusion

The review found significant improvement in loneliness when mindfulness intervention with an average length of 8-week duration was introduced to the population with generally no mental health issue. However, the findings were based on included studies with uncertainty in quality detailed in the review. The review has also identified existing gaps in the literature that investigated the effect of a mindfulness intervention on loneliness with suggestions for future studies to investigate further. Given the current rise in loneliness level, clinicians and the public can consider applying mindfulness intervention to alleviate loneliness when there is no existing mental health condition.

## Data Availability Statement

The original contributions presented in the study are included in the article/Supplementary Material, further inquiries can be directed to the corresponding author/s.

## Author Contributions

ST contributed in data collection and data analysis and manuscript writing. VL and L-HL contributed in data analysis and manuscript writing. All authors contributed to the article and approved the submitted version.

## Conflict of Interest

The authors declare that the research was conducted in the absence of any commercial or financial relationships that could be construed as a potential conflict of interest.
