# Supplementary material for: Can Mindfulness Help to Alleviate Loneliness? A Systematic Review and Meta-Analysis
Source: Front Psychol. 2021 Feb 25;12:633319. doi: 10.3389/fpsyg.2021.633319 (PMC7947335; doi:10.3389/fpsyg.2021.633319)
Supplement: Supplementary file 1 [file Data_Sheet_1.docx]

**Supplemental Table 1. Search Terms Used**

1. Loneliness.mp or exp Loneliness/
2. Lonel*.mp. [mp=abstract, heading words, title]
3. Mindfulness.mp. or exp Mindfulness/
4. Meditation.mp. or exp Meditation/
5. Exp UCLA Loneliness Scale/
6. Exp transcendental meditation/
7. 1 or 2 or 5
8. 3 or 4 or 6
9. 7 and 8

**Supplemental Table 2. Risk of Bias Assessment Version 2.0**

**1) Randomisation**

| **Trials** | **1.1. Sequence random?** | **1.2. Allocation concealed?** | **1.3. Imbalance suggest problem?** |
| --- | --- | --- | --- |
| Creswell 2012 | PY | PN | PN |
| Jazaieri 2012 | PY | PN | PN |
|  | Comment: Method of randomization was not specified. |  |  |
| Dodds 2015 | Y | PN | PN |
|  | Quote: Randomization was performed by the study biostatistician using stratified block randomization using random block size. |  |  |
| Mascaro 2016 | PY | PN | PN |
|  | Comment: Method of randomization not specified. |  |  |
| Zhang 2018 | PY | PN | PN |
|  | Comment: No information |  |  |
| Lee 2019 | Y | Y | PN |
|  | Quote: They were simply randomized into 2 groups by a computer-generated list of random numbers. | Quote: Random allocation sequence was generated by the researchers. Staffs who do not know the detail of the research, enrolled participants and assigned participants to interventions. |  |
| Lindsay 2019 | PY | NI | PN |
|  | Comment: Method of randomization not specified. | |  |
| Pandya 2019 | Y | PN | PN |
|  | Quote: Randomization was done by the researcher using computer generated random number tables | Quote: The random allocation was masked from the potential participants.  Comment: No information for investigator. |  |

NI, No information; PN, Probably no; PY, Probably yes; Y, Yes.

**2) Effect of adhering to intervention**

| **Trials** | **2.1. Were participants aware of their assigned intervention during the trial?** | **2.2. Were carers and people delivering the interventions aware of participants' assigned intervention during the trial?** | **2.3. Were important non-protocol interventions balanced across intervention groups?** | **2.4. Were there failures in implementing the intervention that could have affected the outcome?** | **2.5.** **Was there non-adherence to the assigned intervention regimen that could have affected participants’ outcomes?** | **2.6. If N/PN/NI to 2.3, or Y/PY/NI to 2.4 or 2.5:** **Was an appropriate analysis used to estimate the effect of adhering to intervention?** |
| --- | --- | --- | --- | --- | --- | --- |
| Creswell 2012 | PY | PY | NA | NA | PY | PN |
|  |  |  | Comment: Group session vs wait-list. | Comment: Group session vs wait-list. |  | Comment: Although a per-protocol analysis is performed, it is not considered as an appropriate analysis to account for the adherence issue according to the Revised Cochrane risk-of-bias tool for randomized trials (RoB2). |
| Jazaieri 2012 | PY | PY | PY | PN | PY | NI |
|  |  |  | Comment: Group effects were tried to match between intervention and active control groups. | Comment: The expertise in both intervention and active control groups were probably monitored and standardised. |  |  |
| Dodds 2015 | PY | PY | NA | NA | PY | NI |
|  |  |  | Comment: Group session vs wait-list. | Comment: Group session vs wait-list. |  |  |
| Mascaro 2016 | PY | PY | NA | NA | PY | NI |
|  |  |  | Comment: Group session vs wait-list. | Comment: Group session vs wait-list. |  |  |
| Zhang 2018 | PY | PY | NA | NA | PY | NI |
|  |  |  | Comment: Comparator group not stated. | Comment: Comparator group not stated. |  |  |
| Lee 2019 | PY | PY | PY | PN | PY | NI |
|  |  |  | Comment: Both groups are likely group sessions. | Comment: The expertise in both intervention and active control groups were probably monitored and standardised. |  |  |
| Lindsay 2019 | PY | PY | PY | PN | PY | Y |
|  |  |  | Comment: Both groups were smartphone-based interventions | Comment: The expertise in both groups were probably standardised. |  | Comment: Raw data for baseline and post-intervention were provided for all patients including those who have dropped out. |
| Pandya 2019 | PY | PY | NA | NA | PY | NI |
|  |  |  | Comment: Group session vs no intervention. | Comment: Group session vs no intervention. |  |  |

NA, Not applicable; NI, No information; PN, Probably no; PY, Probably yes; Y, Yes.

**3) Missing outcome data**

| **Trials** | **3.1. Were data for this outcome available for all, or nearly all, participants randomized?** | **3.2. If N/PN/NI to 3.1: Is there evidence that the result was not biased by missing outcome data?** | **3.3. If N/PN to 3.2: Could missingness in the outcome depend on its true value?** | **3.4. If Y/PY/NI to 3.3: Is it likely that missingness in the outcome depended on its true value?** |
| --- | --- | --- | --- | --- |
| Creswell 2012 | PY | NA | NA | NA |
| Jazaieri 2012 | PY | NA | NA | NA |
| Dodds 2015 | PY | NA | NA | NA |
| Mascaro 2016 | Y | NA | NA | NA |
| Zhang 2018 | Y | NA | NA | NA |
| Lee 2019 | PY | NA | NA | NA |
| Lindsay 2019 | Y | NA | NA | NA |
| Pandya 2019 | Y | NA | NA | NA |

NA, Not applicable; PN, Probably no; PY, Probably yes; Y, Yes.

**4) Outcome**

| **Trials** | **4.1. Was the method of measuring the outcome inappropriate?** | **4.2. Could measurement or ascertainment of the outcome have differed between intervention groups?** | **4.3. If N/PN/NI to 4.1 and 4.2: Were outcome assessors aware of the intervention received by study participants?** | **4.4. If Y/PY/NI to 4.3: Could assessment of the outcome have been influenced by knowledge of intervention received?** | **4.5. If Y/PY/NI to 4.4: Is it likely that assessment of the outcome was influenced by knowledge of intervention received?** |
| --- | --- | --- | --- | --- | --- |
| Creswell 2012 | PN | PN | PY | PN (Objective assessment) | N |
| Jazaieri 2012 | PN | PN | PY | PN (Objective assessment) | N |
| Dodds 2015 | PN | PN | PY | PN (Objective assessment) | N |
| Mascaro 2016 | PN | PN | PY | PN (Objective assessment) | N |
| Zhang 2018 | PN | PN | PY | PN (Objective assessment) | N |
| Lee 2019 | PN | PN | PY | PN (Objective assessment) | N |
| Lindsay 2019 | PN | PN | PY | PN (Objective assessment) | N |
| Pandya 2019 | PN | PN | PY | PN (Objective assessment) | N |

PN, Probably no; PY, Probably yes; N, No.

**5) Selection of the reported result**

| **Trials** | **5.1. Were the data that produced this result analysed in accordance with a pre-specified analysis plan that was finalized before unblinded outcome data were available for analysis?** | **Is the numerical result being assessed likely to have been selected, on the basis of the results, from...** | **5.2. ... multiple eligible outcome measurements (e.g. scales, definitions, time points) within the outcome domain?** | **5.3.... multiple eligible analyses of the data?** |
| --- | --- | --- | --- | --- |
| Creswell 2012 | NI |  | PN | PN |
| Jazaieri 2012 | NI |  | PN | PN |
| Dodds 2015 | NI |  | PN | PN |
| Mascaro 2016 | NI |  | PN | PN |
| Zhang 2018 | NI |  | PN | PN |
| Lee 2019 | NI |  | PN | PN |
| Lindsay 2019 | Y |  | PN | PN |
|  | Quote: The study design and outcomes described here were preregistered with Clinical Trials identifier NCT02433431.  Comment: The pre-specified plan was checked. |  |  |  |
| Pandya 2019 | NI |  | PN | PN |

NI, No information; PN, Probably no; PY, Probably yes; Y, Yes.

WMD, (Weighted) Mean difference.

**Supplemental Figure 1. Main Analysis**

**(Varied Mindfulness Interventions in Participants with No Known Mental Health Conditions)**

WMD, (Weighted) Mean difference.

**Supplemental Figure 2. Subgroup Analysis 1**

**(Cognitively-Based Compassion Training Intervention in Participants with No Known Mental Health Conditions)**

SMD, Standardised mean difference.

**Supplemental Figure 3. Subgroup Analysis 2.**

**(Varied Mindfulness Interventions in Participants with Mental Health Conditions)**

SMD, Standardised mean difference.

**Supplemental Figure 4. Subgroup Analysis 3.1**

**(Varied Mindfulness Interventions in Younger Populations Only)**

****SMD, Standardised mean difference.

**Supplemental Figure 5. Subgroup Analysis 3.2.1**

**(Varied Mindfulness Interventions in Adults and Elderly Only;**

**Conservative estimate from Lee 2019)**

SMD, Standardised mean difference.

**Supplemental Figure 6. Subgroup Analysis 3.2.2**

**(Varied Mindfulness Interventions in Adults and Elderly Only)**

WMD, (Weighted) Mean difference

**Supplemental Figure 7. Explorative Analysis 1**

**(Varied Mindfulness Interventions with UCLA-R Loneliness Scale Only)**

SMD, Standardised mean difference.

**Supplemental Figure 8. Explorative Analysis 2**

**(Varied Mindfulness Interventions with UCLA-R Loneliness Scale or UCLA-8 Loneliness Scale)**

SMD, Standardised mean difference.

**Supplemental Figure 9. Explorative Analysis 3**

**(Mindfulness-Based Stress Reduction Intervention Only)**

SMD, Standardised mean difference.

**Supplemental Figure 10. Explorative Analysis 4.1**

**(Trials Employed Loneliness Scales with Increasing Score for Increasing Loneliness;**

**Conservative estimate from Lee 2019)**

SMD, Standardised mean difference.

**Supplemental Figure 11. Explorative Analysis 4.2**

**(Trials with Similar Loneliness Scales That Increasing Score Shows Increasing Loneliness)**
